# Complete genomes of five *Escherichia coli* isolated from focal duodenal necrosis lesions of layer chickens

**DOI:** 10.1128/mra.00646-24

**Published:** 2025-03-25

**Authors:** Yu-Yang Tsai, Julia Ienes-Lima, Yi-Chen Luo, Catherine M. Logue

**Affiliations:** 1 Department of Population Health, College of Veterinary Medicine, University of Georgia 308501, Athens, Georgia, USA; 2 Department of Infectious Diseases, Athens Veterinary Diagnostic Laboratory, University of Georgia 551782, Athens, Georgia, USA; University of Maryland School of Medicine, Baltimore, Maryland, USA

**Keywords:** genomes, *Escherichia coli*, focal duodenal necrosis, layers, poultry

## Abstract

Focal duodenal necrosis (FDN) is an easily overlooked but significant disease of table-egg layers, resulting in reduced egg production. However, the etiology of FDN is poorly understood. Here, we report the complete genomes of five *E. coli* strains isolated from the FDN lesions of laying flocks.

## ANNOUNCEMENT

All five *Escherichia coli* strains were isolated directly from focal duodenal necrosis (FDN) lesions by plating samples of the lesion on blood agar and MacConkey agar with incubation at 37°C for 18–24 h (1). Suspect colonies were identified using 16S rRNA PCR (1, 2) and screened for genes associated with avian pathogenic *E. coli* (APEC) and inflammatory bowel disease (IBD) (3). We found these strains are APEC-like but also carry several IBD-related genes. Among the five FDN strains examined (Table 1), FDN-4 belongs to serogroup O25b:H4 ST131 and shows high genetic similarity to the EC958 strain (4), a strain type that has been associated with urinary tract infections in humans (5). *E. coli* O25:H4 ST131 are also commonly found to harbor fluoroquinolone resistance and extended-spectrum β-lactamase (ESBL) resistance traits (6).

All strains were stored at −80°C in glycerol prior to culture on MacConkey agar. A single colony was picked and grown in Luria–Bertani broth at 37°C for 24 h. The cell suspension was centrifuged, and the cell pellet was shipped to Mr. DNA (www.mrdnalab.com, Shallowater, TX, USA) for whole genome sequencing.

The bacterial pellet of each sample was resuspended in 180 µL of ATL buffer (Qiagen, Germantown, MD) and was used to extract the high molecular weight DNA using MagAttract HMW DNA Kit (Qiagen). The DNA concentration was evaluated using the Qubit dsDNA HS Assay Kit (Life Technologies, Carlsbad, CA), and sample quality was determined using a NANODROP 2000 (ThermoFisher Scientific, Waltham, MA). The size of each gDNA sample was determined using electrophoresis (E-Gel SizeSelect 2% Agarose Gel; Invitrogen), which showed a fluorescent band >15 Kb respectively. The samples were sheared using the Covaris G-tube (Covaris Inc., Woburn, MA), and the average size of each sample verified using the Agilent 2100 Bioanalyzer (Agilent Technologies, Alpharetta, GA). Approximately 1–1.5 µg of gDNA was used as input for library preparation using SMRTbell Express Template Prep Kit 2.0 (Pacific Biosciences, Menlo Park, CA).

During library preparation, the samples underwent DNA damage, end repair, and barcode adapter ligation. Then, libraries were pooled equimolar with 5–6 libraries per pool. Unligated DNA fragments were removed using a nuclease treatment, and following the library preparation, DNA fragments > 7 Kb were selected using the BluePippin automated size-selection instrument (Sage Science Inc, Beverly, MA). The final library concentration was measured by Qubit dsDNA HS Assay Kit (ThermoFisher Scientific), and the average library size was determined by Agilent 2100 BioAnalyzer (Agilent Technologies). The final library was sequenced using the PacBio Sequel IIe (Pacific Biosciences). HiFi Genome assembly was accomplished using the improved phase assembler (IPA) via SMRT Link 10.1.0. The IPA consists of overlapping (pancake), phasing (nighthawk), filtering overlaps (falcon), contig construction (falcon), and polishing (racon). All of the assembly tools were from PacBio (www.pacb.com) and used at the default settings of the software. All five *E. coli* genomes mentioned above were annotated using the NCBI Prokaryotic Genome Annotation Pipeline (PGAP) v. 6.9. Plasmid identification used PlasmidFinder 2.1 with the default setting (7, 8). Default parameters were used for all software unless otherwise specified.

**TABLE 1:** Characteristics of the genome assemblies and annotation statistics

	FDN-4			FDN-9		FDN-11			FDN-24		FDN-50		
GenBank accession no.	CP158026.1			CP158140.1		CP158143.1			CP158147.1		CP158150.1		
SRA accession no.	SRR30200299			SRR30200304		SRR30200308			SRR30200311		SRR30200318		
BioProject accession no.	PRJNA1111845			PRJNA1111857		PRJNA1111859			PRJNA1111864		PRJNA1111872		
Biosample accession no.	SAMN41405104			SAMN41405192		SAMN41405241			SAMN41405320		SAMN41405370		
Total assembly length (bp)	5,192,349			5,035,071		5,061,891			5,034,475		5,206,549		
Total no. of reads (bp)	23,592			29,226		21,656			23,489		25,546		
No. of contigs	4			3		4			3		6		
N50 (bp)	4,966,509			4,798,160		4,845,341			4,797,640		3,554,120		
Chromosome size (bp)	4,966,509			4,798,160		4,845,341			4,797,640		4,921,440		
CDSs (total)	4,972			4,872		4,845			4,873		5,067		
GC content (%)	50.69			50.5		50.61			50.5		50.8		
tRNA	87			86		88			86		87		
Plasmids	3			2		3			2		3		
Plasmid name	pFDN4-1	pFDN4-2	pFDN4-3	pFDN9-1	pFDN9-2	pFDN11-1	pFDN11-2	pFDN11-3	pFDN24-1	pFDN24-2	pFDN50-1	pFDN50-2	pFDN50-3
Accession no.	CP158027.1	CP158028.1	CP158029.1	CP158141.1	CP158142.1	CP158144.1	CP158145.1	CP158146.1	CP158148.1	CP158149.1	CP158151.1	CP158152.1	CP158153.1
Plasmid size (bp)	182,828	35,593	7,419	148,663	88,248	135,559	48,395	32,596	148,663	88,172	158,974	89,593	36,542
Plasmidfinder/plasmidtype	IncFIB/IncFIC	IncX1	Not identified	IncFIB/IncFII	IncI1-I	IncFIB/IncFIC	Not identified	IncX1	IncFIB/IncFII	IncI1-I	IncFIB/IncFII	IncI1-I	IncX4

## Data Availability

The genome sequences of the five *E. coli* FDN associated strains were deposited in GenBank under the accession numbers shown in the Table 1.
